# Comprehensive *In Silico* Analysis of a Novel Serum Exosome-Derived Competitive Endogenous RNA Network for Constructing a Prognostic Model for Glioblastoma

**DOI:** 10.3389/fonc.2021.553594

**Published:** 2021-03-05

**Authors:** Zihao Wang, Xin Ji, Lu Gao, Xiaopeng Guo, Wei Lian, Kan Deng, Bing Xing

**Affiliations:** ^1^ Department of Neurosurgery, Peking Union Medical College Hospital, Chinese Academy of Medical Sciences and Peking Union Medical College, Beijing, China; ^2^ China Pituitary Disease Registry Center, Chinese Pituitary Adenoma Cooperative Group, Beijing, China; ^3^ Department of Nuclear Medicine, The First Affiliated Hospital of Nanjing Medical University, Jiangsu Province Hospital, Nanjing, China

**Keywords:** glioblastoma, exosome, liquid biopsy, ceRNA, prognostic model

## Abstract

**Purpose:**

Glioblastoma (GBM) is one of the most aggressive brain tumors with high mortality, and tumor-derived exosomes provide new insight into the mechanisms of GBM tumorigenesis, metastasis and therapeutic resistance. We aimed to establish an exosome-derived competitive endogenous RNA (ceRNA) network for constructing a prognostic model for GBM.

**Methods:**

We obtained the expression profiles of long noncoding RNAs (lncRNAs), miRNAs, and mRNAs from the GEO and TCGA databases and identified differentially expressed RNAs in GBM to construct a ceRNA network. By performing lasso and multivariate Cox regression analyses, we identified optimal prognosis-related differentially expressed lncRNAs (DElncRNAs) and generated a risk score model termed the exosomal lncRNA (exo-lncRNA) signature. The exo-lncRNA signature was subsequently validated in the CGGA GBM cohort. Finally, a novel prognostic nomogram was constructed based on the exo-lncRNA signature and clinicopathological parameters and validated in the CGGA external cohort. Based on the ceRNA hypothesis, oncocers were identified based on highly positive correlations between lncRNAs and mRNAs mediated by the same miRNAs. Furthermore, regression analyses were performed to assess correlations between the expression abundances of lncRNAs in tumors and exosomes.

**Results:**

A total of 45 DElncRNAs, six DEmiRNAs, and 38 DEmRNAs were identified, and an exosome-derived ceRNA network was built. Three optimal prognostic-related DElncRNAs, HOTAIR (HR=0.341, P<0.001), SOX21-AS1 (HR=0.30, P<0.001), and STEAP3-AS1 (HR=2.47, P<0.001), were included to construct the exo-lncRNA signature, which was further proven to be an independent prognostic factor. The novel prognostic nomogram was constructed based on the exo-lncRNA signature, patient age, pharmacotherapy, radiotherapy, IDH mutation status, and MGMT promoter status, with a concordance index of 0.878. ROC and calibration plots both suggested that the nomogram had beneficial discrimination and predictive abilities. A total of 11 pairs of prognostic oncocers were identified. Regression analysis suggested excellent consistency of the expression abundance of the three exosomal lncRNAs between exosomes and tumor tissues.

**Conclusions:**

Exosomal lncRNAs may serve as promising prognostic predictors and therapeutic targets. The prognostic nomogram based on the exo-lncRNA signature might provide an intuitive method for individualized survival prediction and facilitate better treatment strategies.

## Introduction

Glioma is one of the most aggressive brain tumors, and it has received considerable attention due to its relatively high incidence, poor prognosis and significant impact on quality of life. Glioblastoma (GBM, WHO Grade IV) is the most common type of glioma, accounting for 56.6% of all glioma cases, and has an average annual incidence of 3.21 per 100,000 people in the United States (1). GBM remains difficult to treat, with a median survival of 12-15 months regardless of aggressive surgical resection, radiotherapy or concomitant chemotherapy, and the 5-year survival rate is reported to be only 5.6% (1, 2). In recent years, an increasing number of molecularly targeted therapies have emerged but have not achieved satisfactory outcomes due to the complex pathogenesis and molecular heterogeneity of GBM. More studies are needed to explore the mechanism involved and to identify biomarkers to predict prognosis and therapeutic outcomes of GBM.

Exosomes are nanosized (30-150 nm) extracellular vesicles released by various cell types and are present in the blood and other body fluids, allowing for noninvasive analyses in real time (3, 4). It is recognized that exosomes can regulate the bioactivity of the recipient cell by transferring molecular and genetic cargo, including proteins, lipids, and small RNAs (3). Recent studies have highlighted the role of exosomes in tumors, including tumor progression, metastasis, establishment of the tumor microenvironment, and drug resistance (5–7). The importance of exosomes and their cargo (especially small RNAs) for GBM has gradually been realized, as is the case for microRNAs (miRNAs) in regulating angiogenesis and tumor metastasis (8) and messenger RNAs (mRNAs) in mediating cell migration and drug resistance (9, 10). Nonetheless, integration analysis of the exosome expression profile in GBM has not been fully elucidated.

Because multiple signaling pathways and genes are interrupted in tumor pathogenesis, target genes might be masked by other unnecessary genes. The competitive endogenous RNA (ceRNA) hypothesis provides a systemic perspective to explore potential exosome-derived biomarkers to predict GBM diagnosis and prognosis. The ceRNA network hypothesizes that crosstalk between RNAs, including long noncoding RNAs (lncRNAs), mRNAs and miRNAs, forms large-scale regulatory networks through shared miRNA response elements (MREs) (11). Among them, lncRNAs act as an endogenous competitive molecule sponges that bind to miRNAs through MREs and further regulates mRNA expression. Considerable studies have confirmed the role of the lncRNA-miRNA-mRNA regulation network, and based on the ceRNA hypothesis, oncocers that play crucial roles in oncogenic pathways have been identified in various cancers, such as lung cancer, prostate cancer, and liver cancer (12–15). These results suggest the significance of the ceRNA network in the comprehensive analysis of gene interactions and the identification of potential biomarkers for tumor diagnosis, therapy, and prognosis.

In this study, we aimed to investigate and validate an exosome-derived multiple gene expression signature based on a ceRNA network that can predict prognosis and provide potential targets for GBM treatment. Furthermore, based on the exosome signature and clinical factors, we constructed a promising GBM prognostic nomogram model with beneficial predictive ability and accuracy.

## Materials and Methods

### Data Retrieval and Processing

Datasets including quantified gene expression profiles of exosomes in GBM were obtained from the Gene Expression Omnibus (GEO) database (http://www.ncbi.nlm.nih.gov/geo/) (16). Only two datasets were found: GSE106804 (lncRNA/mRNA) and GSE112462 (miRNA). GSE106804, based on the GPL18573 platform (Illumina NextSeq 500), contains 13 GBM serum exosome samples and 6 normal serum exosome samples from healthy donors. GSE112462, based on the GPL24781 platform (Nanostring human miRNA panel), contains 10 GBM serum exosome samples and 8 normal samples from healthy donors. Furthermore, we extracted information on the gene expression profiles in GBM tumor tissues and normal brain tissues from TCGA (lncRNA/mRNA), GSE80338 (lncRNA/mRNA), GSE63319 (miRNA) and GSE25631 (miRNA). We selected patients with complete clinical and survival data from the cohort of TCGA and ultimately included 151 GBM patients to form a training set with TCGA data. The validation set was formed based on the Chinese Glioma Genome Atlas (CGGA, http://www.cgga.org.cn) database, including 350 GBM patients. Because the data were extracted from GEO, TCGA, and CGGA, approval for our study by the ethics committee was waived.

### Identification of Differentially Expressed RNAs

For normalized gene expression profile data, we used edgeR version 3.24.3 to screen DE-RNAs between GBM serum exosomes and normal serum exosomes, using GSE106804 for differentially expressed lncRNAs (DElncRNAs)/DEmRNAs and GSE112462 for DEmiRNAs. A log fold change ≥ 2 and false discovery rate (FDR) < 0.01 were considered as the screening criteria (17). These DE-RNAs are illustrated in volcano plots. We also analyzed the DE-RNAs between GBM tumor tissues and normal brain tissues *via* a similar approach, with TCGA and GSE80338 for DElncRNAs/DEmRNAs and GSE63319 and GSE25631 for DEmiRNAs. Subsequently, the DE-RNAs obtained from these two steps were intersected to screen final DElncRNAs, DEmiRNAs, and DEmRNAs from both GBM exosome samples and tumor samples, and the results are depicted in Venn diagrams.

### Construction of the Competitive Endogenous RNA Network

Interactions between DElncRNAs and DEmiRNAs were predicted using the miRcode database (http://www.mircode.org/) (18). DEmRNAs targeted by DEmiRNAs were retrieved from the databases TargetScan, miRTarBase, and miRDB (19–21). Only the mRNAs in the miRNA-mRNA relationship pairs recognized in all 3 databases were selected as candidate genes for constructing the ceRNA network, as based on previously identified lncRNA-miRNA and miRNA-mRNA relationship pairs. The network was visualized using Cytoscape software (https://cytoscape.org/).

### Functional and Pathway Enrichment Analyses

Functional enrichment analysis of the DEmRNAs in the ceRNA network was performed using Database for Annotation, Visualization, and Integrated Discovery (DAVID, https://david.ncifcrf.gov/) (22), an online functional annotation tool used for Gene Ontology (GO) functional enrichment and Kyoto Encyclopedia of Genes and Genomes (KEGG) pathway enrichment analyses (23, 24). A P value < 0.05 was considered statistically significant.

### Construction and Evaluation of the Prognostic Risk Score Model Based on Exosomal Differentially Expressed Long Noncoding RNAs

lncRNAs exhibit great species, tissue, and cell specificity and play a dominant role in the upstream part of the ceRNA network, which affects the function of mRNAs or miRNAs. Thus, lncRNAs might be optimal biomarkers for GBM diagnosis as well as prognosis evaluation. To identify survival-associated exo-lncRNAs, univariate Cox regression was first performed using the survival package in R 3.5.1 (http://bioconductor.org/packages/survival/) (25); DElncRNAs with a P value < 0.05 were selected for further lasso regression analysis. Optimal prognosis-related exo-lncRNAs were identified using multivariate Cox regression analysis based on the Akaike information criterion (AIC) and used to construct a prognostic risk score model for predicting overall survival (OS) (26). The formula of the risk score model was as follows: risk score = expression level of Gene_1_ × β_1_ + expression level of Gene_2_ × β_2_ +…+ expression level of Gene_n_ × β_n_, where β is the regression coefficient calculated by multivariate Cox regression analysis (27). Using the median risk score as the cutoff value, patients were divided into high- and low-risk groups (27).

Kaplan-Meier (K-M) survival curves were constructed to estimate the prognosis of high-risk and low-risk patients, and the survival differences between these two groups were assessed by a two-sided log-rank test. The predictive and distinguishing ability of the risk score model within 0.5, 1, 2, and 3 years was evaluated using Harrell’s concordance index (C-index) and time-dependent receiver operating characteristic (ROC) curve analysis in the R package ‘survcomp’ (http://www.bioconductor.org/packages/survcomp/) and ‘survivalROC’ (https://cran.r-project.org/web/packages/survivalROC/) (28). The values of the C-index and area under the ROC curve (AUC) range from 0.5 to 1, with 1 indicating perfect discrimination and 0.5 indicating no discrimination. The exo-lncRNA-based prognostic model constructed by the cohort from TCGA was validated in the GBM cohort from CGGA using a similar method.

### Associations Between the Exosomal Long Noncoding RNA Signature and Tumor Immune Microenvironment

The tumor immune microenvironment (TIME) patterns and immunogenomic features of GBM were assessed, and the associations between TIME and exo-lncRNA signature were further analyzed. Estimation of Stromal and Immune cells in Malignant Tumor tissues using Expression data (ESTIMATE) was used to evaluate the overall patterns of tumor microenvironment based on the gene expression profiles of GBM samples (29). The abundances of intratumoral stromal cells (stromal score) and immune cells (immune score), and the tumor purity were predicted by ESTIMATE algorithm. In addition, 31 immune signatures, introduced by He et al, was utilized to represent the overall immune activity of tumors, including the types, functions and molecular pathways of tumor infiltrating immune cells (TIICs) (30). The enrichment levels of those immune gene sets were quantified by single-sample gene set enrichment analysis (ssGSEA), and then compared between high- and low-risk group (31). Furthermore, Pearson correlation analysis was performed to assess the associations between exo-lncRNA signature and the enrichment levels of 31 immune signatures. P < 0.05 and Pearson correlation coefficient > 0.3 were considered statistically significant.

### Construction and Validation of the Nomogram

Univariate Cox regression and multivariate Cox regression analyses were performed with TCGA training set and CGGA validation set data to detect whether clinical characteristics are significantly associated with OS in GBM patients. All independent prognostic factors were then selected to construct a prognostic nomogram using the rms R package in the cohort from TCGA.

The discrimination ability of the nomogram was quantitatively evaluated by the C-index and the AUC (28). In addition, calibration plots were performed to graphically evaluate the discriminative ability of the nomogram (28). To compare the ability of the two prognostic models, the exo-lncRNA signature and the nomogram, in predicting OS, we calculated the net reclassification improvement (NRI) index by using the ‘PredictABEL’ package in R. Finally, the prognostic nomogram was externally validated using the CGGA cohort. All analyses were performed with R version 3.5.1, and a two-tailed P value of < 0.05 was considered statistically significant. Hazard ratios (HRs) and 95% confidence intervals (CIs) are reported if necessary.

### Identification of Prognostic Oncocers of Glioblastoma

Oncocers, as defined by ceRNA-mediated cross-talk by sponging miRNAs in oncogenesis, were identified on the basis of the ceRNA hypothesis, demonstrating positive correlations between lncRNAs and mRNAs mediated by the same miRNAs (11, 15). Pearson correlation analysis and regression analysis were applied to identify prognostic oncocers of GBM based on the three prognostic-related lncRNAs and the ceRNA network. P < 0.05 and cor (Pearson correlation coefficient) > 0.3 were considered statistically significant. Furthermore, Pearson correlation tests were performed to assess the expression abundance of lncRNAs in tumors and exosomes.

### Gene Set Enrichment Analysis of Differentially Expressed Long Noncoding RNAs

Setting the expression level of a lncRNA as the population phenotype, GSEA (http://software.broadinstitute.org/gsea/index.jsp) was performed to identify related KEGG pathways and molecular mechanisms of exosomal DElncRNAs in the risk score model and oncocers (31). Enriched gene sets with a nominal P value < 0.05 and an FDR q value < 0.25 were considered statistically significant.

## Results

### Identification of Differentially Expressed mRNAs, Differentially Expressed Long Noncoding RNAs, and Differentially Expressed miRNAs in Glioblastoma Exosomes

**Figure 1:**
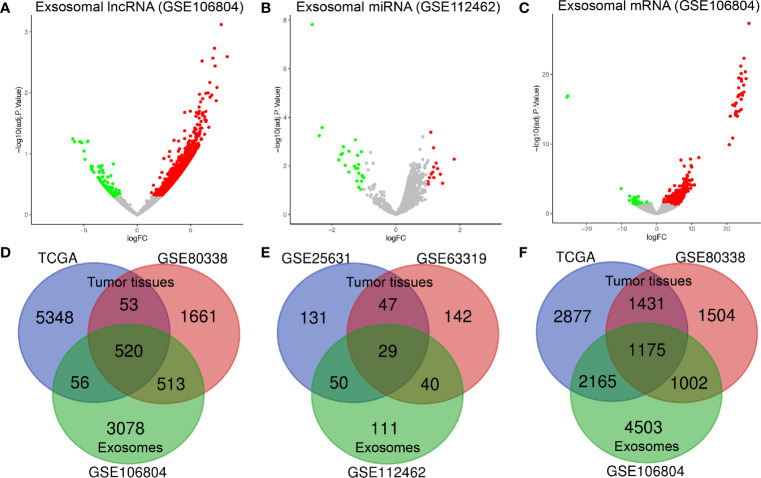
Identification of differentially expressed exosome-related genes. Volcano plots of **(A)** DElncRNAs, **(B)** DEmiRNAs, and **(C)** DEmRNAs between GBM serum exosomes and normal samples. The red dots represent upregulated genes, and the green dots represent downregulated genes. The Venn diagram shows **(D)** 520 DElncRNAs, **(E)** 29 DEmiRNAs, and **(F)** 1175 DEmRNAs in the overlapping part of exosome-related DE-RNAs and GBM tissue-related DE-RNAs. adj. P value: adjusted P value; FC: fold change.

By applying the screening criteria, 4167 DElncRNAs, 230 DEmiRNAs, and 8845 DEmRNAs were identified between GBM serum exosomes and normal serum exosomes and are displayed in the volcano plots (**Figures 1A–C**). In terms of the DE-RNAs from GBM tumor samples and normal brain samples, we obtained 573 DElncRNAs and 2606 DEmRNAs from TCGA and GSE80338 and 76 DEmiRNAs from GSE25631 and GSE63319. Finally, we obtained 520 DElncRNAs, 29 DEmiRNAs, and 1175 DEmRNAs from both GBM tumor samples and serum exosomes (**Figures 1D–F**).

### Construction of the Competitive Endogenous RNA Network

**Figure 2:**
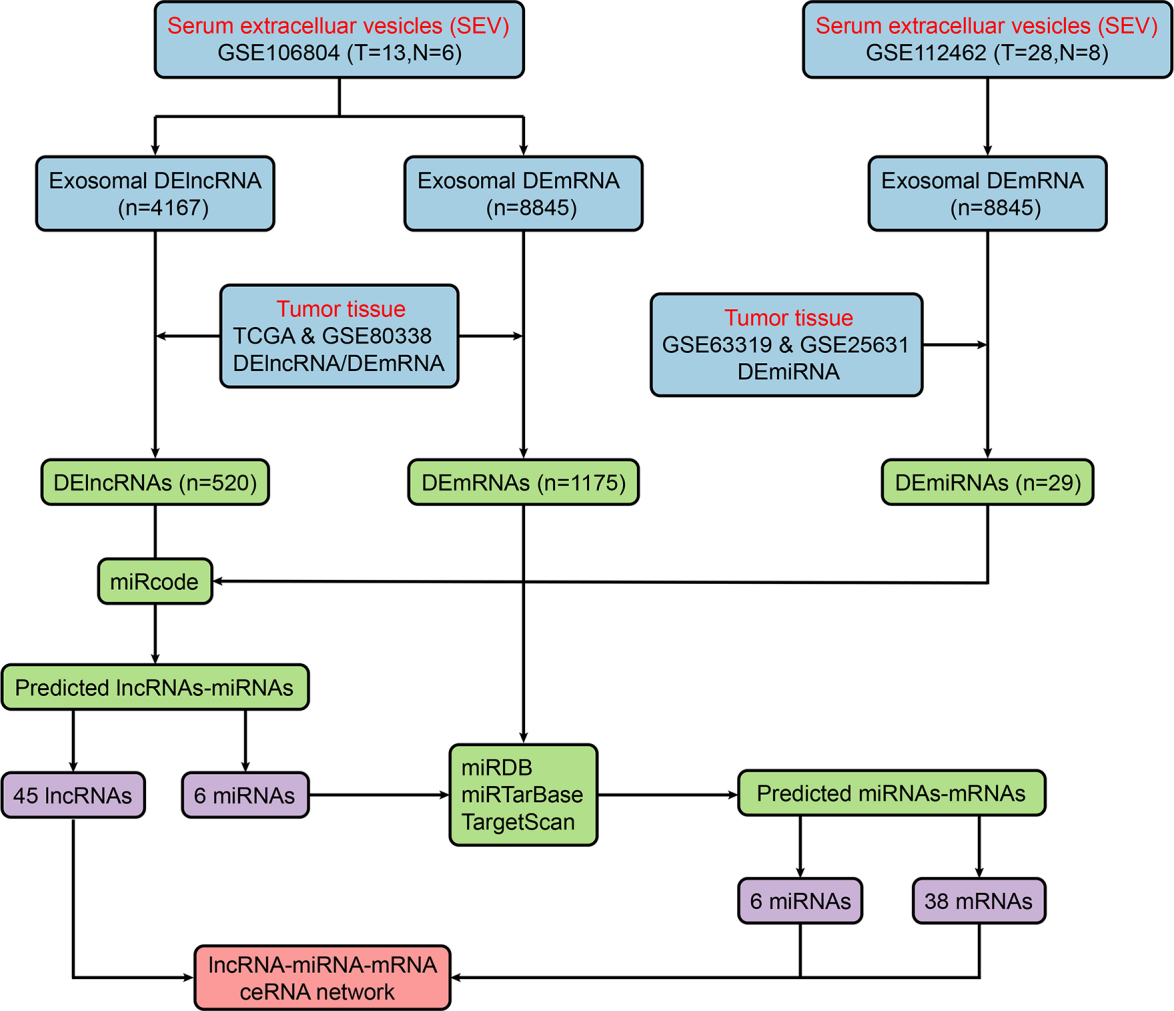
Flow chart of ceRNA network construction. ceRNA, competitive endogenous RNA; DElncRNA, differentially expressed long noncoding RNA; miRDB, miRNA database; miRNA, microRNA; mRNA, messenger RNA.

**Figure 3:**
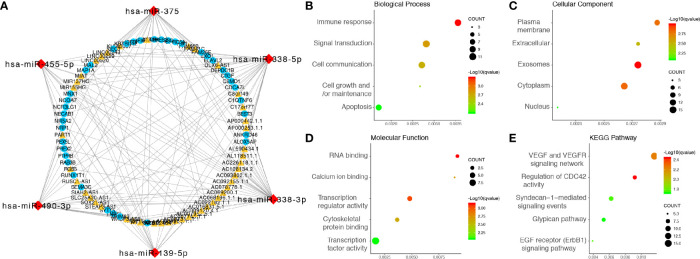
Integrated analysis of the ceRNA network. **(A)** ceRNA network, the yellow triangles represent 45 lncRNAs, the red diamonds represent six miRNAs, and the blue dots represent 38 mRNAs. **(B)** Biological processes enriched in DE-RNAs. **(C)** CCs enriched in DE-RNAs. **(D)** MFs enriched in the DE-RNAs. **(E)** KEGG pathways enriched in the DE-RNAs. KEGG, Kyoto Encyclopedia of Genes and Genomes.

**Figure 2** shows the flow chart of the construction of the ceRNA network. We first predicted potential miRNAs that interact with 520 DElncRNAs through the miRcode database. Next, we selected intersecting genes between the predicted miRNAs and 29 DEmiRNAs to obtain lncRNA-miRNA interaction pairs comprising 45 lncRNAs and six miRNAs. To improve the reliability of the bioinformatics prediction, the abovementioned six miRNAs were input into the TargetScan, miRTarBase and miRDB databases to identify common mRNAs. These common mRNAs were compared to the 1175 DEmRNAs to identify intersecting components, and miRNA-mRNA interaction pairs of six miRNAs and 38 mRNAs were obtained. Ultimately, 45 lncRNAs, six miRNAs and 38 mRNAs were incorporated to construct the ceRNA network, which was visualized with Cytoscape (**Figure 3A**).

### Gene Ontology and Kyoto Encyclopedia of Genes and Genomes Enrichment Analyses

GO analysis, involving biological process (BP), cellular component (CC) and molecular function (MF) categories, was performed with the 38 mRNAs in the newly formed ceRNA network. For BP categories, DEmRNAs were significantly enriched in the terms immune responses, signal transduction and cell communication (**Figure 3B**). In the CC category, DEmRNAs were significantly enriched in the terms plasma membrane, extracellular and exosome (**Figure 3C**). In addition, DEmRNAs were significantly enriched in the MF category terms RNA binding, calcium ion binding and transcription regulator activity (**Figure 3D**). Moreover, KEGG pathway analysis revealed that DEmRNAs were mainly enriched in the VEGF and VEGFR signaling networks, the regulation of CDC42 activity and Syndecan-1-mediated signaling events (**Figure 3E**).

### Construction and Evaluation of the Prognostic Risk Score Model Based on Exosomal Differentially Expressed Long Noncoding RNAs (exo-lncRNA Signature)

Univariate Cox regression analysis was performed on the 45 candidate DElncRNAs, 28 of which correlated significantly with OS. We then applied lasso‐penalized Cox regression to select potential prognosis-related DElncRNAs, revealing six lncRNAs (**Supplementary Figure 1**). Multivariate Cox regression analysis was performed, and three lncRNAs were confirmed, namely, HOTAIR, SOX21-AS1, and STEAP3-AS1, which were included in the construction of the prognostic risk score model (exo-lncRNA signature). The final prognostic risk score formula was as follows: risk score = expression level of HOTAIR × 0.341 + expression level of SOX21-AS1 × (-1.208) + expression level of STEAP3-AS1 × 0.903.

**Figure 4:**
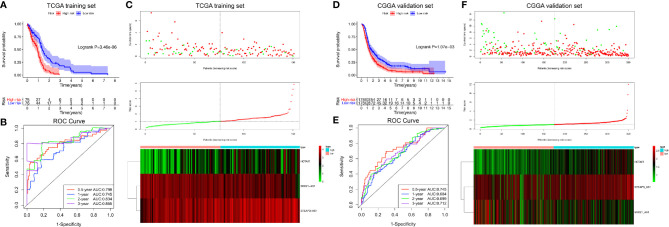
Risk score analysis of the GBM exo-lncRNA signature in the training set from TCGA and validation set from CGGA. **(A–C)** Analysis performed using the training set from TCGA; **(D–F)** CGGA validation set analysis. **(A, D)** Kaplan-Meier survival curve of high-risk and low-risk groups. The table below shows the number of patients at risk at different time points. **(B, E)** Time‐dependent ROC curves based on risk score level. **(C, F)** Risk score analysis of the GBM exo-lncRNA signature. Upper panel: patient survival status and time distributed by risk score. Middle panel: risk score curve of the exo-lncRNA signature. Bottom panel: heatmap of SOX21-AS1, HOTAIR, and STEAP3-AS1 expression in GBM samples. The colors from red to green indicate expression level from high to low. The dotted line indicates the individual inflection point of the risk score curve, by which the patients were categorized into low-risk and high-risk groups. ROC: receiver operating characteristic.

Next, we calculated the prognostic score of each patient in the training set from TCGA and used the median risk score as the cutoff value to classify all patients into high-risk (high-risk score, 75 patients) and low-risk (low-risk score, 76 patients) groups (**Figure 4C**). K-M survival analysis showed that the high-risk group had significantly poorer OS than the low-risk group (log-rank P = 3.46×10^-6^), and the 0.5-, 1-, 2-, and 3-year OS rates of the high-risk group versus (vs) the low-risk group were 65.6% vs 82.6%, 43.4% vs 71.6%, 8.1% vs 32.9%, and 0.0% vs 18.1%, respectively (**Figure 4A**). The C-index of the exo-lncRNA signature was 0.831 (95% CI, 0.801-0.861). In addition, the exo-lncRNA signature showed favorable predictive ability for 0.5-, 1-, 2-, and 3-year OS rates in the training set from TCGA, with AUC values of 0.798, 0.745, 0.834, and 0.855, respectively (**Figure 4B**).

To confirm the adequacy of the prognostic exo-lncRNA signature in different populations, an external validation set from CGGA was applied. A total of 350 patients in the CGGA dataset were classified into a low-risk group (175 patients) and a high-risk group (175 patients) by using the median risk score as the cutoff (**Figure 4F**), and the OS of the high-risk group was significantly lower than that of the low-risk group (log-rank P = 1.07×10^-3^; **Figure 4D**). The AUC values of survival prediction within 0.5, 1, 2, and 3 years in the CGGA validation set were 0.745, 0.684, 0.699, and 0.712, respectively, suggesting the favorable predictive ability of the exo-lncRNA signature (**Figure 4E**).

Finally, combined survival analyses were performed to investigate the roles of the exo-lncRNA signature in predicting the OS of patients with or without standard chemoradiotherapy, namely, concurrent temozolomide (TMZ) and radiotherapy. As displayed in **Supplementary Figures 2A, B**, patients receiving standard chemoradiotherapy showed significantly better OS than did those who did not receive standard chemoradiotherapy. Notably, patients treated with standard chemoradiotherapy, whether high or low risk, commonly exhibited better outcomes than did those without standard chemoradiotherapy in both cohorts from TCGA and CGGA (**Supplementary Figures 2C, D**). Interestingly, high-risk patients receiving standard chemoradiotherapy would have a better OS than low-risk patients.

### Associations Between Exosomal Long Noncoding RNA Signature and Tumor Immune Microenvironment

**Figure 5:**
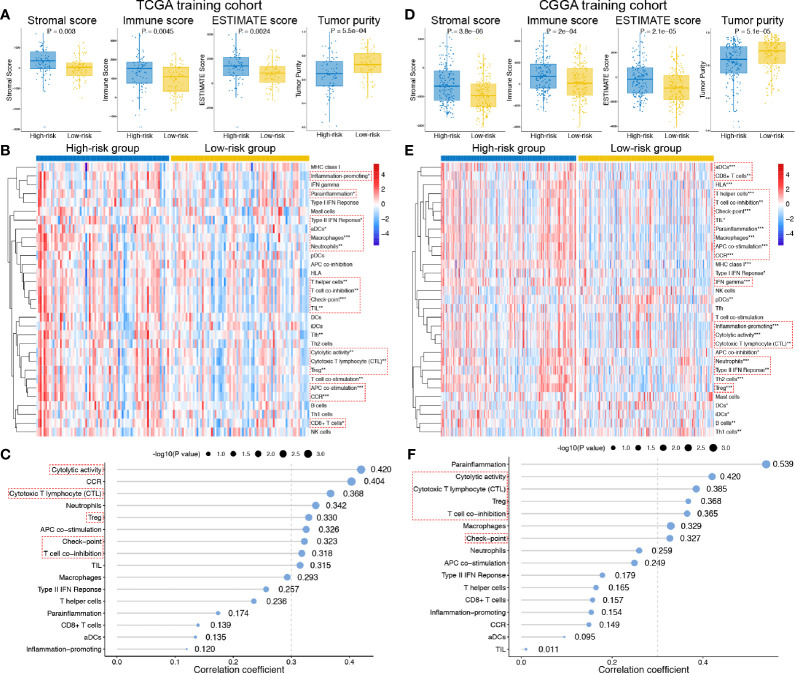
Associations between the exo-lncRNA signature and tumor immune microenvironment in the TCGA training set and CGGA validation set. **(A, C)** Comparisons of the infiltration level of stromal and immune cells, the ESTIMATE score and tumor purity between high- and low-risk group by boxplots. **(B, E)** Comparisons of the ssGSEA scores of 31 immune signatures between high- and low-risk group by heatmap. Red dotted boxes indicated 16 intersected immune signatures in both training and validation cohort. *means P < 0.05, **means P < 0.01, and ***means P < 0.001. **(C, F)** Correlation analysis between the exo-lncRNA signature and 16 immune signatures. Red dotted boxes indicated five intersected immune signatures in both training and validation cohort.

The general TIME patterns of GBM patients were firstly assessed by the ESTIMATE algorithm, and the immune, stromal, and ESTIMATE scores were significantly higher in the high-risk group, indicating higher infiltration levels of immune and stromal cells in the high-risk GBM patients in both training and validation cohort (**Figures 5A, D**). In contrast, tumor purity was lower in the high-risk group in both cohorts (**Figures 5A, D**). Additionally, the enrichment levels of the 31 immune signatures, representing the overall immune activity of GBM, were quantified by ssGSEA. Eighteen immune signatures significantly differed between high- and low-risk group in the TCGA training set (**Figure 5B**), and 27 immune signatures differed between two groups in the CGGA validation set (**Figure 5E**), and a total of 16 intersected immune signatures (red dotted box) were selected for further analysis. Then, correlation analyses between the exo-lncRNA signature and 16 immune signatures were analyzed, and nine immune signatures were significantly correlated with the exo-lncRNA signature in the TCGA cohort (**Figure 5C**), and seven signatures in the CGGA cohort (**Figure 5F**). Finally, the intersected five immune signatures, including cytolytic activity, cytotoxic T lymphocyte level, regulatory T cell, checkpoint molecules, and T cell co-inhibition, were believed to be significantly positively correlated with the exo-lncRNA-based risk score.

### Determination of the Exosomal Long Noncoding RNA Signature as an Independent Prognostic Factor

The demographics and clinicopathological characteristics of GBM patients in the training set from TCGA and validation cohort from CGGA based on the exo-lncRNA signature are shown in **Table 1**. Univariate Cox regression analysis was first performed to detect whether the exo-lncRNA signature or clinicopathological factors are significantly associated with OS in the training set from TCGA (**Table 2**). The exo-lncRNA signature (P = 2.48×10^-8^), age (P = 1.98×10^-4^), pharmacotherapy (P = 1.06×10^-4^), radiotherapy (P = 1.04×10^-3^), IDH mutation status (P = 8.91×10^-3^), MGMT promoter status (P = 6.84×10^-3^), and ATRX status (P = 4.28×10^-2^) were found to be significantly associated with OS. Multivariate Cox regression analysis was subsequently performed to identify independent prognostic factors (**Table 2**), revealing the exo-lncRNA signature (P = 1.33×10^-4^), age (P = 1.45×10^-2^), pharmacotherapy (P = 1.55×10^-2^), radiotherapy (P = 3.21×10^-4^), IDH mutation status (P = 2.31×10^-2^), and MGMT promoter status (P = 1.33×10^-2^). Therefore, the prognostic exo-lncRNA signature constructed by the training set from TCGA was observed to be an independent prognostic factor for GBM, and it was confirmed to be an independent prognostic factor in the external validation cohort from CGGA (**Table 2**).

**Table 1:** Demographics and clinicopathological characteristics of GBM patients in TCGA training and CGGA validation cohorts based on the exo-lncRNA signature.

Variables	TCGA training cohort			CGGA validation cohort		
	Total (n=151)	Low risk (n=76)	High risk (n=75)	Total (n=350)	Low risk (n=175)	High risk (n=175)
**Age (years)**	59.6 ± 13.7	57.7 ± 14.4	61.6 ± 12.7	48.1 ± 13.3	48.0 ± 12.6	48.1 ± 14.0
**Sex**						
**Female**	53	25	28	139	70	69
**Male**	98	51	47	211	105	106
**KPS**						
**< 80**	32	14	18	NA		
**>= 80**	81	42	39	NA		
**NA**	38	20	18	NA		
**Pharmacotherapy**						
**TMZ**	64	32	32	61 (No)	23	38
**TMZ+BEV**	26	13	13	269 (Yes)	139	130
**Others (No TMZ)**	19	8	11	–	–	–
**No or NA**	42	23	19	20 (NA)	13	7
**Radiotherapy**						
**No**	22	9	13	48	17	31
**Yes**	122	65	57	283	147	136
**NA**	7	2	5	19	11	8
**Surgery**						
**Biopsy only**	16	9	7	NA		
**Tumor resection**	135	67	68	NA		
**IDH status**						
**Wildtype**	147	68	79	270	114	156
**Mutant**	8	8	0	80	61	19
**MGMT promoter status**						
**Methylated**	66	29	37	NA		
**Unmethylated**	85	47	38	NA		
**TERT status**						
**Wildtype**	146	73	73	NA		
**Mutant**	5	3	2	NA		
**BRAF status**						
**Wildtype**	146	73	73	NA		
**Mutant**	5	3	2	NA		
**ATRX status**						
**Wildtype**	140	66	74	NA		
**Mutant**	11	10	1	NA		
**EGFR status**						
**Wildtype**	97	50	47	NA		
**Mutant**	54	26	28	NA		
**1p/19q status**						
**Noncodeletion**	NA			323	153	170
**Codeletion**	NA			17	15	2
**NA**	NA			10	7	3

GBM, glioblastoma; NA, not available; KPS, Karnofsky performance score; TMZ, temozolomide; BEV, bevacizumab; PCV, procarbazine lomustine vinCRISTine.

“Others (No TMZ)” in pharmacotherapy included PCV, PCV+BEV, and other drugs, including avastin, carmustine, and irinotecan. Bold type means P < 0.05.

**Table 2:** Univariate and multivariate Cox proportional hazards analysis of clinicopathological variables and exo-lncRNA signature in TCGA GBM training and CGGA GBM validation cohorts.

Variables	TCGA training cohort (N=151)				CGGA validation cohort (N=350)			
	Univariate Analysis		Multivariate analysis		Univariate Analysis		Multivariate analysis	
	HR (95% CI)	P value	HR (95% CI)	P value	HR (95% CI)	P value	HR (95% CI)	P value
**Age**	1.028(1.013–1.044)	**1.98e-04**	1.017(1.002–1.012)	**1.45e-02**	1.078(1.048–1.108)	**8.35e-05**	1.017(1.001–1.033)	**1.17e-02**
**Sex**	0.916(0.626–1.341)	0.65	–	–	1.063(0.837–1.350)	0.61	–	–
**KPS**	0.926(0.696–1.233)	0.59	–	–	NA		NA	
**Pharmacotherapy**	0.883(0.852–0.913)	**1.06e-04**	0.951(0.936–0.966)	**1.55e-02**	0.573(0.432–0.759)	**1.04e-04**	0.603(0.444–0.819)	**1.20e-03**
**Radiotherapy**	0.433(0.262–0.714)	**1.04e-03**	0.343(0.192–0.615)	**3.21e-04**	0.668(0.492–0.908)	**9.96e-03**	0.879(0.863–0.895)	**4.76e-02**
**Surgery**	0.934(0.523–1.667)	0.82	–	–	NA		NA	
**IDH mutation status**	0.262(0.096–0.715)	**8.91e-03**	0.141(0.026–0.764)	**2.31e-02**	0.752(0.566–0.988)	**3.89e-02**	0.856(0.840–0.872)	**3.34e-02**
**MGMT promoter status**	1.434(1.133–1.733)	**6.84e-03**	1.250(1.235–1.265)	**1.33e-02**	NA		NA	
**TERT promoter status**	0.906(0.287–2.861)	0.87	–	–	NA		NA	
**BRAF status**	1.973(0.720–5.410)	0.19	–	–	NA		NA	
**ATRX status**	0.426(0.187–0.973)	**4.28e-02**	0.899(0.734–3.942)	0.08	NA		NA	
**EGFR status**	1.273(0.873–1.857)	0.21	–	–	NA		NA	
**1p/19q status**	NA		NA		0.913 (0.662–1.259)	0.58	–	–
**Exo-lncRNA signature**	1.733(1.428–2.102)	**2.48e-08**	1.785(1.326–2.403)	**1.33e-04**	1.394(1.280–1.518)	**1.94e-14**	1.385(1.262–1.521)	**6.78e-12**

OS, overall survival; GBM, glioblastoma; NA, not available; HR, hazard ratio; CI, confidence interval; KPS, Karnofsky performance score; TMZ, temozolomide; BEV, bevacizumab; PCV, procarbazine lomustine vinCRISTine.

“Others (no TMZ)” in pharmacotherapy included PCV, PCV+BEV, and other drugs, including avastin, carmustine, and irinotecan.

All statistical tests were two-sided. Bold type means P < 0.05.

### Construction and Validation of the Prognostic Nomogram

**Figure 6:**
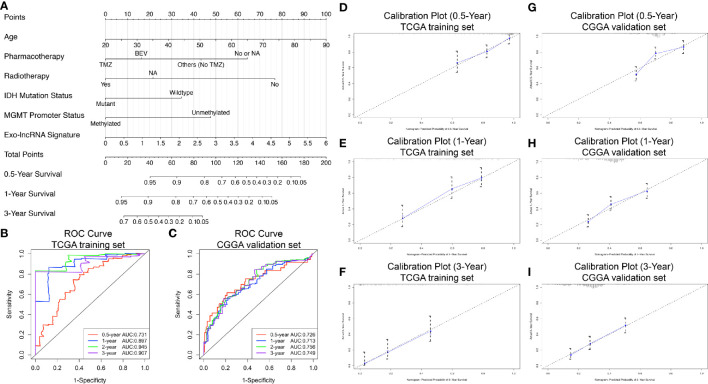
Nomogram to predict the 0.5-, 1-, and 3-year survival probability of patients with GBM. Prognostic nomogram **(A)** to predict the survival of GBM patients based on the training set from TCGA. Time‐dependent ROC curves based on risk score level in training set from TCGA **(B)** or in the validation cohort from CGGA **(C)**. Calibration curves of the nomogram for predicting survival at 0.5, 1, and 3 years in the training set from TCGA **(D–F)** and the validation cohort from CGGA **(G–I)**. The X-axis indicates the nomogram-predicted probability, and the y-axis indicates the actual survival.

We constructed a prognostic nomogram using the six independent prognostic factors identified to provide a clinically applicable method for prognosis prediction in GBM patients. **Figure 6A** shows the established nomogram that predicted survival probability at 0.5, 1, and 3 years based on the training set from TCGA. The C-index of the nomogram was 0.878 (95% CI, 0.847–0.909). The AUC values of the 0.5-, 1-, 2-, and 3-year survival prediction using the nomogram were 0.731, 0.897, 0.945, and 0.907, respectively, which indicated a favorable predictive ability (**Figure 6B**). Additionally, the calibration plots (**Figures 6D–F**) showed consistency between the nomogram prediction and actual observation with regard to the 0.5-, 1-, and 3-year survival rates in the cohort from TCGA. NRI analysis indicated that the proportions of correct reclassification of the nomogram increased by 20.3% in 1 year (P < 0.001) and 21.5% in 2 years (P < 0.001). These results suggest that the predictive ability of the nomogram at 1 and 2 years is significantly better than that of the exo-lncRNA signature (**Supplementary Figure 3A**).

In addition, based on the CGGA external validation cohort, the C-index of the nomogram in predicting OS was 0.746 (95% CI, 0.718–0.776), and the AUC values and calibration plots both demonstrated great prediction performance for 0.5-, 1-, and 3-year OS in the cohort from CGGA (**Figures 6C, G–I**). Finally, NRI analysis in the CGGA cohort also demonstrated significant improvements in the NRI index of the nomogram at 1, 2, and 3 years compared with the exo-lncRNA signature (**Supplementary Figure 3B**).

### Identification of Prognostic Oncocers of Glioblastoma

**Figure 7:**
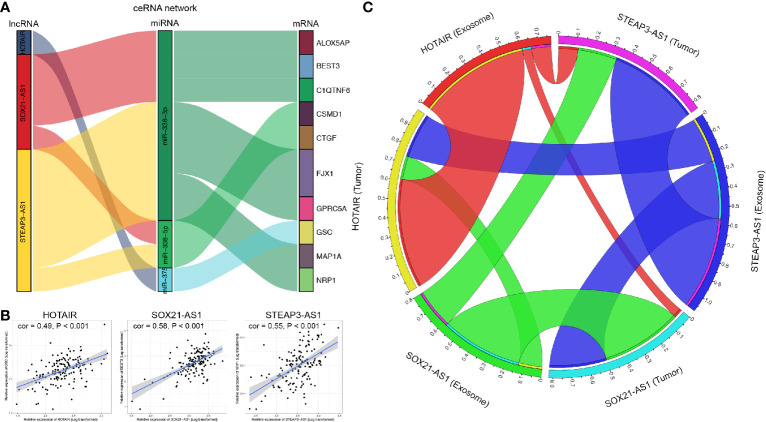
Correlation analysis of lncRNAs and mRNAs. **(A)** Identified lncRNA-miRNA-mRNA axes. Left bar: lncRNA; middle bar: miRNA; right bar: mRNA. **(B)** lncRNAs versus protein-coding genes as indicated. The gray area around the blue line represents the 95% CI. **(C)** Correlation analysis of lncRNAs between GBM exosomes and tumor tissue. lncRNA, long noncoding RNA; miRNA, microRNA; mRNA, messenger RNA.

Based on the ceRNA network and the three prognostic-related lncRNAs, we further performed regression analysis and Pearson’s correlation tests to identify prognostic oncocers; P < 0.05 and cor > 0.3 were considered to indicate statistical significance (**Figure 7A**, **Supplementary Table 1**). A total of 11 pairs of prognostic oncocers were identified. GSC, BEST3, and NRP1 were the most significant correlative mRNAs for HOTAIR, SOX21-AS1, and STEAP3-AS1, respectively (**Figure 7B**). Positive correlations were also obtained for these lncRNA-mRNA pairs. miR-338-3p and miR-338-5p are involved in multiple ceRNA pathways, as shown in **Figure 7A**. Furthermore, regression analysis suggested excellent consistency of the expression abundance of the 3 prognostic-related lncRNAs between exosomes and tumor tissue, with 0.61 (P<0.001), 0.45 (P<0.001), and 0.51 (P<0.001) for HOTAIR, SOX21-AS1, and STEAP3-AS1, respectively, as illustrated in **Figure 7C**.

### Expression Analysis, Survival Analysis, and Gene Set Enrichment Analysis of the Three Prognosis-Related Long Noncoding RNAs

**Figure 8:**
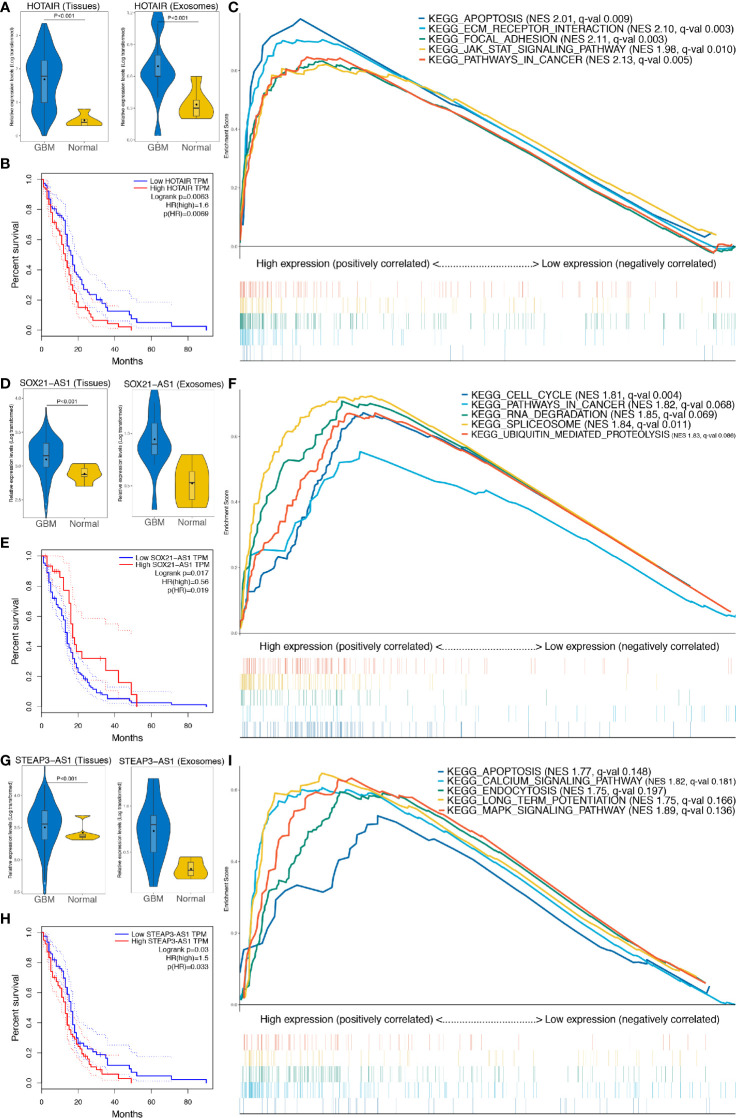
Further analysis of the three lncRNAs included in the exo-lncRNA signature. **(A, D, G)** Expression analysis of HOTAIR, SOX21-AS1, and STEAP3-AS1 in GBM exosomes and tissues. **(B, E, H)** Survival analysis of HOTAIR, SOX21-AS1, and STEAP3-AS1, respectively. **(C, F, I)** GSEA of HOTAIR, SOX21-AS1, and STEAP3-AS1, respectively.

Expression of HOTAIR, SOX21-AS1, and STEAP3-AS1 in GBM tissue or exosomes was significantly higher than that in normal samples (P < 0.001), as shown in **Figures 8A, D, G**, respectively. In addition, survival analyses confirmed that high expression of HOTAIR and STEAP3-AS1 is related to a worse OS, with high expression of SOX21-AS1 being associated with a better OS (**Figures 8B, E, H**). According to GSEA, the high HOTAIR, SOX21-AS1, and STEAP3-AS1 expression groups are mainly enriched in pathways related to cancer, including apoptosis, ECM receptor interaction, focal adhesion, the JAK-STAT signaling pathway, cancer, cell cycle, cancer, the calcium signaling pathway, and the MAPK signaling pathway (**Figures 8C, F, I**, **Supplementary Table 2**).

## Discussion

GBM is one of the most aggressive brain tumors with high mortality and poor prognosis. The high recurrence rate after surgery and therapeutic resistance of GBM have prompted researchers to further explore the molecular mechanism and new therapeutic targets. Tumor-derived exosomes provide new insight into the mechanisms of tumorigenesis, metastasis and therapeutic resistance. Studies have demonstrated that exosomes released by GBM contain cargo, such as EGFRvIII, VEGF, and miR21, that are selectively enriched for oncogenic-related functions and are transferred to recipient cells to stimulate tumor proliferation, angiogenesis, and invasion (32, 33). In addition, exosomes have been reported to contribute to therapeutic resistance, and treatments targeting exosomes have proven effective. For example, Chuang et al. (34) found that STAT3 inhibitors could overcome temozolomide resistance by downregulating miR-21-enriched exosomes in GBM. All these studies suggest that exosomes may serve as biomarkers for diagnosis, prognosis, and treatment in GBM.

With the development of high-throughput genome-sequencing technologies and the availability of different open databases, numerous studies have performed in silico analysis to identify novel biomarkers for GBM. Indeed, the ceRNA hypothesis helped us to better understand the crosstalk between RNAs and more comprehensively analyze the complex gene interactions underlying cancerogenesis. Previous studies have proposed multiple gene expression patterns in GBM, and these patterns might predict prognosis and guide treatment (35, 36). Although several studies have constructed ceRNA networks and found meaningful biomarkers (37–39), exosome-derived ceRNA networks in GBM have not been constructed to date.

Our study first identified shared DElncRNAs, DEmiRNAs, and DEmRNAs by comparing normal serum exosomes or brain tissues with GBM serum exosomes or tumor tissues based on the GEO and TCGA databases. After predicting lncRNA-miRNA interactions and miRNA-mRNA interactions, 45 lncRNAs, six miRNAs and 38 mRNAs were chosen for constructing the ceRNA network, which provided an integrated view of the crosstalk between GBM-specific transcripts. In addition, this ceRNA network improved the prediction accuracy of potential candidate biomarkers for prognosis and treatment, as it narrowed the scope of research.

Due to evolutionarily nonconserved characteristics, lncRNAs display high species, tissue, and cell specificity and are thus optimal biomarkers for GBM. We identified three exosomal lncRNAs (HOTAIR, SOX21-AS1, and STEAP3-AS1) that correlated significantly with prognosis in the ceRNA network through lasso and multivariate Cox regression analyses. Further survival analysis confirmed high expression of HOTAIR and STEAP3-AS1 and low expression of SOX21-AS1 to be associated with a low OS rate, indicating the potential predictive ability of these three exo-lncRNAs. HOTAIR is expressed by a sequence within the highly conserved HOX gene, which has been found to be highly expressed in multiple tumors, including breast cancer, ovarian cancer, esophageal cancer, and colorectal cancer (40–44). HOTAIR participates in cell growth and promotes tumor development and metastasis (42). In addition, HOTAIR is able to reprogram the chromatin state to promote cancer metastasis in a polycomb repressive complex 2 (PRC2)-dependent manner (44). Consistent with our results, Zhou et al. (45) found that high expression of HOTAIR is related to poor survival outcome and that depletion of HOTAIR inhibits GBM cell migration or invasion, indicating that HOTAIR is a potential therapeutic target. SOX21-AS1 is also reported to be associated with multiple cancers, though with great heterogeneity among tumors. SOX21-AS1 has been verified as an oncogene in tumor progression, as it is highly expressed in lung adenocarcinoma, hepatocellular carcinoma, and colorectal cancer and predicts poor prognosis (46–48). Conversely, Yang et al. (49) discovered that low expression of SOX21-AS1 was significantly associated with poor clinicopathological features and prognosis in oral cancer patients, proposing that SOX21-AS1 might reinforce other tumor suppressor mRNAs and might otherwise competitively bind to certain transcription factors, thus preventing oncogene transcription. It was also suggested that SOX21-AS1 significantly suppresses cervical tumorigenesis (50). Moreover, Paul et al. (51). revealed that lncRNA SOX21-AS1 plays a protective role in glioblastoma, as shown by our results. The different roles of SOX21-AS1 in different cancers indicate its crucial clinical value and prognostic predictive ability. STEAP3-AS1 is less well understood than HOTAIR or SOX21-AS1, and it has only been reported to be associated with a poor OS in tongue squamous cell carcinoma (52).

Based on HOTAIR, SOX21-AS1, and STEAP3-AS1, we further constructed and validated a novel exo-lncRNA signature to calculate risk scores in GBM. We found that patients with high-risk scores presented with significantly poorer OS than did patients with low-risk scores, suggesting that the former patients might need more aggressive treatments and more regular follow-up examinations to detect occurrence. In addition, subsequent univariate and multivariate Cox regression analyses identified that this novel signature can independently predict OS in GBM patients. All these findings demonstrate the important value of this exo-lncRNA signature in clinical decision making.

For more intuitive application in clinical work, we established a novel prognostic nomogram with favorable predictive ability that incorporates the exo-lncRNA signature and clinical parameters, including age, pharmacotherapy, radiotherapy, IDH mutation status, and MGMT promoter status. Subsequently, TCGA/CGGA-based calibration plots indicated its excellent predictive performance. To our knowledge, this is the first nomogram incorporating a serum exosome-derived ceRNA network for predicting GBM patient prognosis, providing a convenient method to perform individualized survival prediction and improve treatment strategies.

Furthermore, we performed correlation analyses between DElncRNAs and DEmRNAs in the ceRNA network and verified the positive correlations between lncRNAs and mRNAs mediated by the same miRNAs. Interestingly, we found that miR-338-3p and miR-338-5p are involved in multiple ceRNA pathways, indicating their role in GBM pathogenesis. Previous studies have also elucidated that miR-338-3p/5p is involved in the development of GBM and other tumors. For example, miR-338-3p inhibits GBM proliferation by targeting MAP4K3 and suppresses angiogenesis by inhibiting EGFL7 (53, 54); miR338-5p was also found to enhance tumor metastasis by inhibiting TSHZ3 expression and thus promoting MMP2 expression (55). Lei et al. (56). proposed that miR-338-5p suppresses GBM proliferation and metastasis by inhibiting EFEMP1, and miR-338-5p reportedly sensitizes GBM cells to radiotherapy by regulating genes involved in the DNA lesion response (57). Thus, miR-338-3p/5p might serve as a therapeutic target for GBM.

Our study still has some limitations. First, clinicopathological information of the GBM cohorts extracted from online databases (TCGA and CGGA) was inevitably limited. In addition, detailed information such as neuroimaging and resection extent was not included in the nomogram due to incomplete data. Second, previous studies have found that tumor-associated myeloid cells (TAMCs), accounting for approximately 50% of the GBM mass, play pivotal roles in the progression and chemotherapy resistance of GBM (58). These TAMCs might also release exosomes in addition to GBM cells. In our study, exosomes from the GSE106804 dataset were tumor specific because they were obtained through a sensitive analytical microfluidic platform (EVHB-Chip). However, GSE112462 used Exoquick plus to detect exosomes, which may be from both GBM tumor cells and TAMCs. Regardless of whether exosomes originate from tumor cells or TAMCs, crosstalk exists in those cells and exosomes, and both contribute to tumorigenesis and progression and are associated with prognosis (59). Thus, the principal findings of our study still provide a convincing and robust prognostic model for GBM. Third, experimental validation *in vivo* and *in vitro* was not carried out due to the in silico nature of the study, though the results of the present study may serve as a foundation for further experiments on cell lines or clinical samples. Finally, further validation of the prognostic nomogram in large-scale prospective clinical cohorts is needed.

In conclusion, a serum exosome-derived ceRNA regulation network associated with lncRNAs was successfully constructed, providing insight into the crosstalk among various RNA transcripts. We identified a reliable exosome-related three-lncRNA risk score model that can independently predict prognosis and provide potential therapeutic targets for GBM. We also established a novel promising prognostic nomogram model based on the exo-lncRNA signature and clinical parameters that may facilitate the prediction of GBM prognosis and guide individualized treatment.

## Data Availability Statement

Publicly available datasets were analyzed in this study. These data can be found here: Gene Expression Omnibus (GEO, http://www.ncbi.nlm.nih.gov/geo/), Chinese Glioma Genome Atlas (CGGA, http://www.cgga.org.cn), The Cancer Genome Atlas (TCGA, https://portal.gdc.cancer.gov/).

## Author Contributions

LG and XG performed the data curation and analysis. KD and WL analyzed and interpreted the results. XJ, ZW, and BX drafted and reviewed the manuscript. All authors contributed to the article and approved the submitted version.

## Funding

This study was supported by the Graduate Innovation Fund of the Chinese Academy of Medical Sciences and Peking Union Medical College (2019–1002–73) and the China Postdoctoral Science Foundation (2019 M650567).

## Conflict of Interest

The authors declare that the research was conducted in the absence of any commercial or financial relationships that could be construed as a potential conflict of interest.
