# Supplementary material for: Comprehensive In Silico Analysis of a Novel Serum Exosome-Derived Competitive Endogenous RNA Network for Constructing a Prognostic Model for Glioblastoma
Source: Front Oncol. 2021 Mar 5;11:553594. doi: 10.3389/fonc.2021.553594 (PMC7973265; doi:10.3389/fonc.2021.553594)
Supplement: Supplementary file 1 [file DataSheet_1.docx]

**Supplementary Figure Legends**

**Supplementary Figure 1. Lasso-penalized and multivariate Cox regression analysis of 28 DElncRNAs.** Lasso regression analysis was further applied for feature selection. The survival-associated DElncRNAs obtained from the univariate analysis were further screened by Lasso regression analysis, and then the remaining molecules were inputted into multivariate analysis to construct the final prognostic model. **(A)** Ten-fold cross-validation was used to calculate the best lambda, which leads to minimum mean crossvalidated error. Red dots represent partial likelihood deviance; solid vertical lines indicate their corresponding 95% CI; the left dotted vertical line is the value of lambda that gives minimum cvm; the right dotted vertical line is the largest value such that error is within 1 standard error of the minimum. **(B)** The coefficient values at varying levels of penalty; each curve represents a lncRNA. **(C)** Multivariate Cox regression analysis, SOX21-AS1, HOTAIR, and STEAP3-AS1 were the three prognosis-related lncRNAs finally identified, which were included in the construction of the Exo-lncRNA signature. CI: confidence interval; coef, regression coefficient; HR, hazard ratio.

**Supplementary Figure 2. Survival analysis of the patients with or without standard chemoradiotherapy.** Kaplan-Meier survival curve of the patients with or without standard chemoradiotherapy in the TCGA training set **(A)** and CGGA validation set **(B)**. Combined survival analysis of exo-lncRNA signature and standard chemoradiotherapy in the TCGA training set **(C)** and CGGA validation set **(D)**. GBM patients with standard chemoradiotherapy, whether high-risk or low-risk, commonly demonstrated better outcomes than those without standard chemoradiotherapy.

**Supplementary Figure 3. Net reclassification improvement (NRI) analysis was performed to compare the OS predictive ability of the exo-lncRNA signature and the nomogram in the TCGA training set (A) and CGGA validation set (B).** NRI > 0 and P < 0.05 means positive improvement of the new model (the prognostic nomogram), NRI < 0 and P < 0.05 means negative improvement of the new model, and NRI = 0 or P > 0.05 means no improvement of the new model.
