# Supplementary figures and images for: Comprehensive In Silico Analysis of a Novel Serum Exosome-Derived Competitive Endogenous RNA Network for Constructing a Prognostic Model for Glioblastoma
Source: Front Oncol. 2021 Mar 5;11:553594. doi: 10.3389/fonc.2021.553594 (PMC7973265; doi:10.3389/fonc.2021.553594)

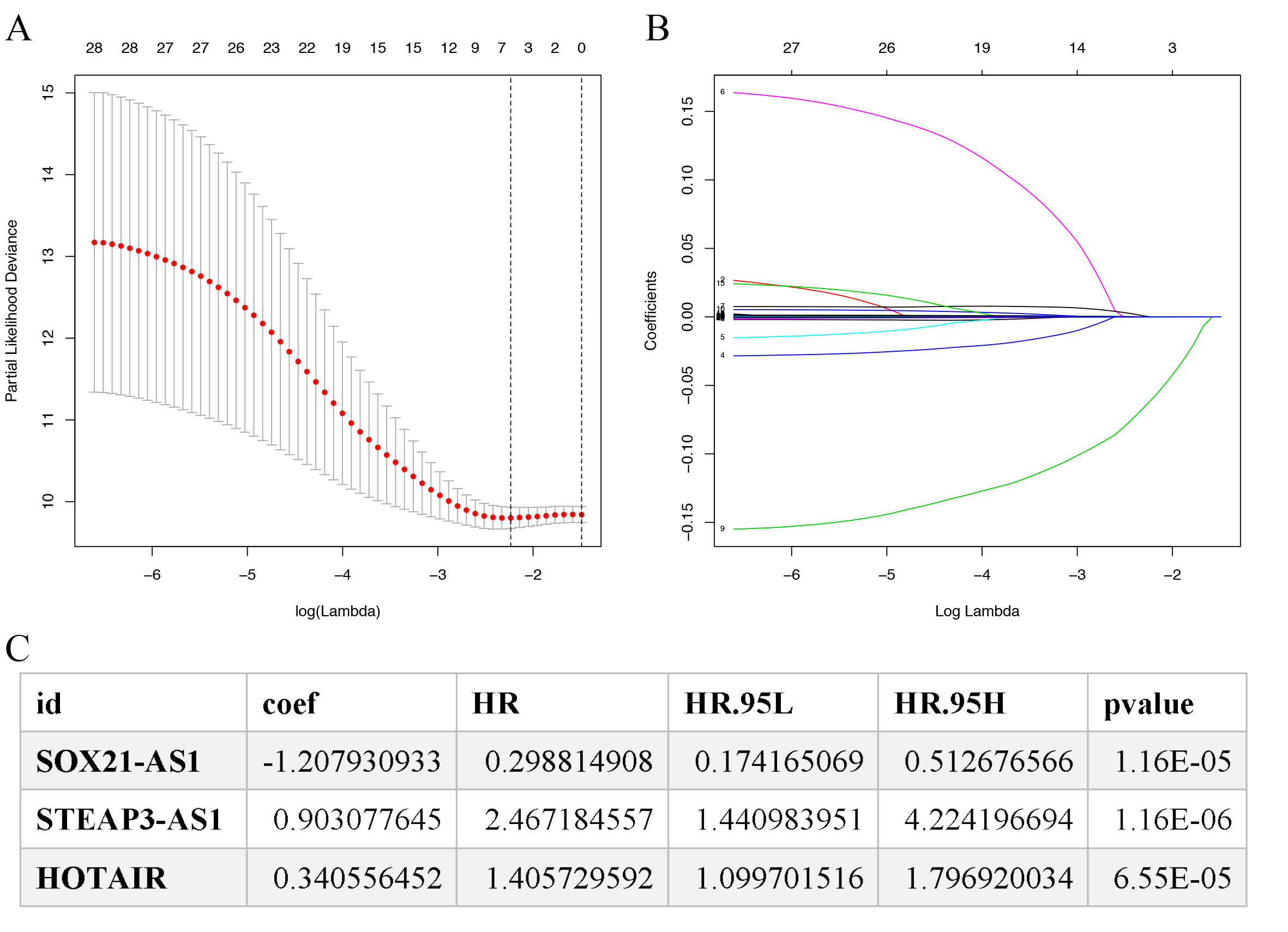

Supplement: Supplementary file 2 [file Image_1.tif]

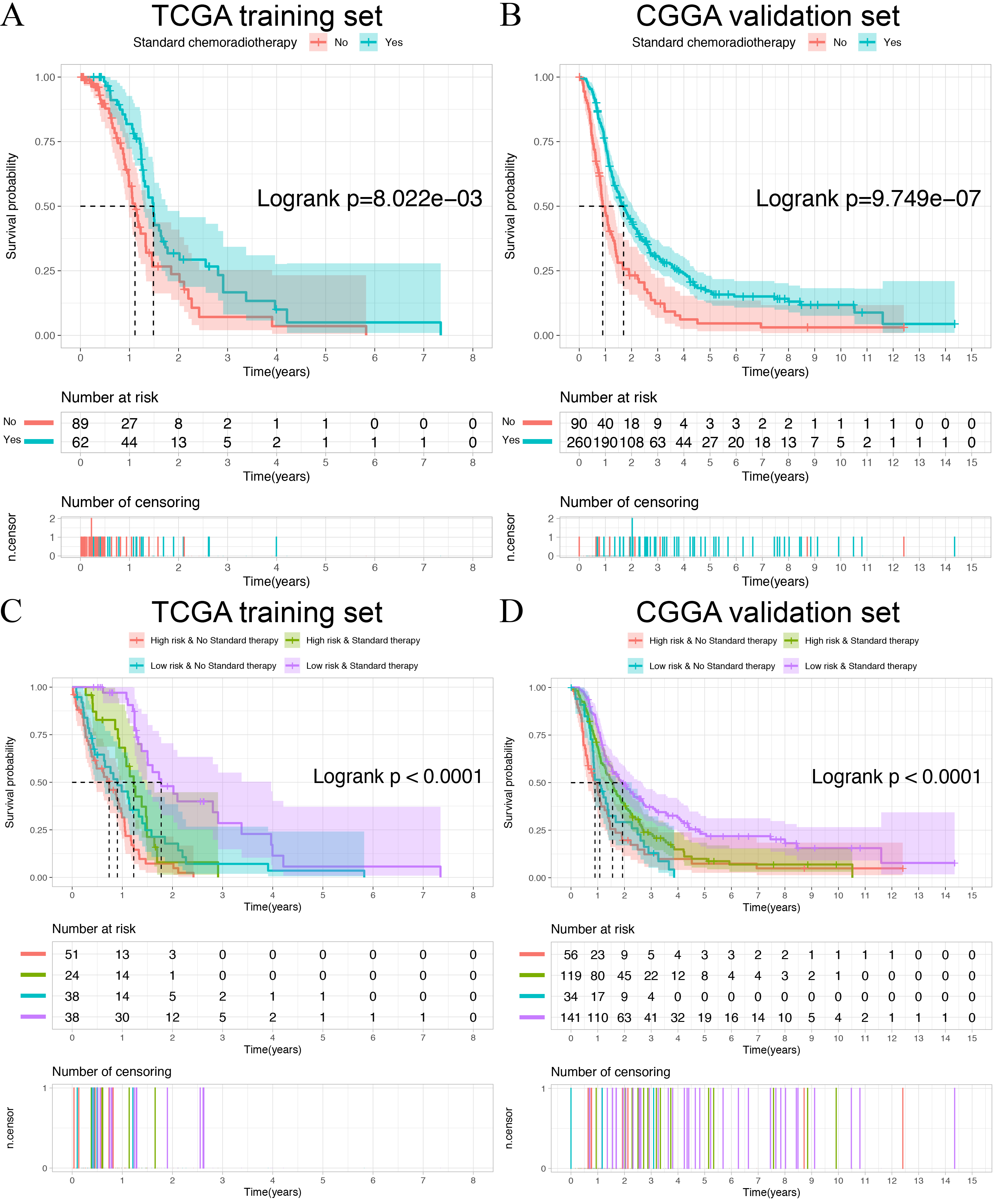

Supplement: Supplementary file 3 [file Image_2.tif]

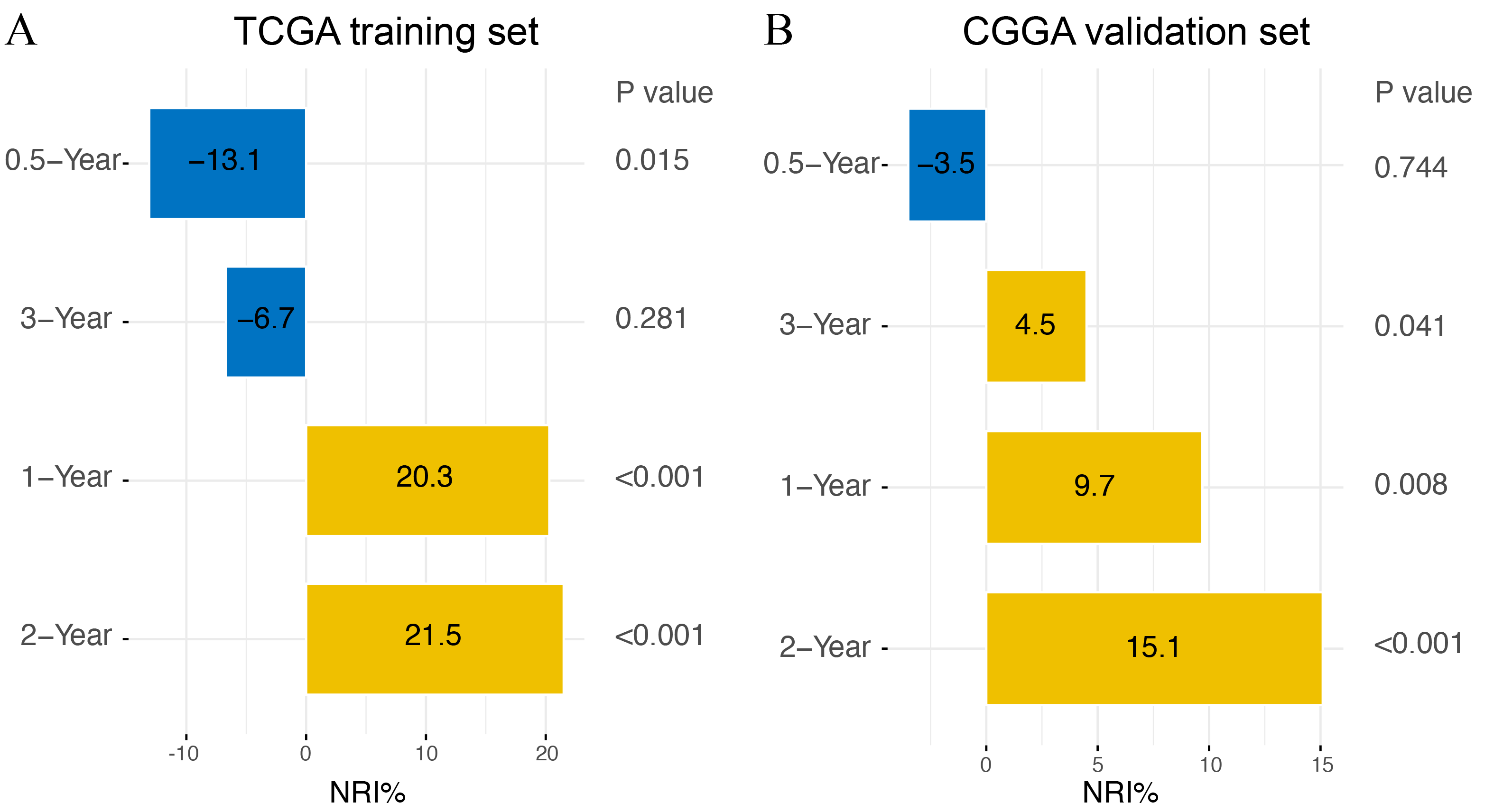

Supplement: Supplementary file 4 [file Image_3.tif]
